# What do meta-analysts need in primary studies? Guidelines and the SEMI checklist for facilitating cumulative knowledge

**DOI:** 10.3758/s13428-024-02373-9

**Published:** 2024-04-16

**Authors:** Belén Fernández-Castilla, Sameh Said-Metwaly, Rodrigo S. Kreitchmann, Wim Van Den Noortgate

**Affiliations:** 1 Faculty of Psychology, Universidad Nacional de Educación a Distancia, Juan del Rosal 10, 28040 Madrid, Spain; 2 Faculty of Psychology and Educational Sciences, KU, Leuven, Belgium; 3 Imec-Itec, KU, Leuven, Belgium; 4 Faculty of Education, Damanhour University, Damanhour, Egypt

**Keywords:** Meta-analysis, Cumulative knowledge, Checklist

## Abstract

Meta-analysis is often recognized as the highest level of evidence due to its notable advantages. Therefore, ensuring the precision of its findings is of utmost importance. Insufficient reporting in primary studies poses challenges for meta-analysts, hindering study identification, effect size estimation, and meta-regression analyses. This manuscript provides concise guidelines for the comprehensive reporting of qualitative and quantitative aspects in primary studies. Adhering to these guidelines may help researchers enhance the quality of their studies and increase their eligibility for inclusion in future research syntheses, thereby enhancing research synthesis quality. Recommendations include incorporating relevant terms in titles and abstracts to facilitate study retrieval and reporting sufficient data for effect size calculation. Additionally, a new checklist is introduced to help applied researchers thoroughly report various aspects of their studies.

Meta-analysis is a statistical technique that emerged in response to the need to combine results from studies addressing similar research questions to draw a general conclusion about the state-of-the-art of a given research topic (Glass, 1976). This methodology began to be implemented in the 1980s when it was uncommon for authors to make the datasets utilized in their studies freely available. The difficulty in accessing raw data led to the need to use the results reported in each study to obtain a quantitative measure of the strength of the effect of interest, namely the effect size measure (Glass et al., 1981; Ray & Shadish, 1996).

The introduction of meta-analysis as a research synthesis technique has led to several potential advantages. Meta-analyses rely on replicable, transparent, and inclusive methodology to identify relevant studies (encompassing not only peer-reviewed results but also pertinent gray literature; Rytwinski et al., 2021). By accumulating data from multiple studies, a meta-analysis allows for more accurate estimation of the overall effect size, maximizing the statistical power and generalizability of the effect size, assessing heterogeneity across studies and explaining it through moderator variables, answering questions not researched in individual studies, developing hypotheses for future consideration, and permitting a regular update of results using newly available data (Deeks et al., 2008; Egger & Smith, 1997; Haidich, 2010; Walker et al., 2008). For these reasons, meta-analysis is frequently considered the highest rank in the hierarchy of evidence (Cooper et al., 2019), implying greater trust in its results than in those of primary studies. This underscores the importance of ensuring that the results of meta-analyses are as reliable and valid as possible.

While meta-analysis is a valuable methodology, it poses a significant challenge due to the considerable time it demands. The process involves searching, screening, and extracting data from all relevant studies, calculating effect sizes and corresponding sampling variances, and carrying out statistical analyses (i.e., syntheses of effect sizes and meta-regression analyses, Cooper et al., 2019). Each step is time-consuming, and complications arise when relevant primary study information is not (clearly) reported. For instance, if the variables of interest are expressed in ambiguous terms in the title or abstract, meta-analysts might have difficulties retrieving that study. Also, insufficient reporting of key study characteristics (e.g., related to sample, design, or setting) hinders meta-analysts’ ability to extract relevant information and incorporate it into meta-regression analyses. Primary researchers might also fail to report quantitative information essential for meta-analysts to calculate effect sizes. Hence, proper reporting of various aspects of primary studies can facilitate more efficient work for meta-analysts, leading to thorough and rigorous research syntheses. Since primary researchers may not always be aware of the information required by a meta-analyst for integrating their study into a research synthesis, the goal of this manuscript is to offer concise instructions on reporting both qualitative and quantitative aspects of primary research. This will enable primary researchers to improve the eligibility of their studies for inclusion in future research syntheses, ultimately resulting in heightened visibility and impact within the academic community and society.

Numerous guidelines for conducting and reporting quantitative research are available and endorsed (e.g., Appelbaum et al., 2018). Adhering to these guidelines can enhance the overall quality of a study. However, it must be noted that improved quality does not necessarily guarantee eligibility for inclusion in a meta-analysis. Meta-analysis criteria often involve additional considerations beyond individual study quality, emphasizing factors such as data relevance and sufficiency. Therefore, meeting guidelines is a valuable step, but researchers should be mindful of the distinct requirements for meta-analytic eligibility. In addressing this issue, Chow et al. (2023) introduced guidelines, with a strong focus on open science. While our study incorporates several of their guidelines, we also introduce supplementary ones not covered by Chow et al. (2023). For instance, we emphasize the role of thorough reporting in aiding various steps of a meta-analysis, including study searching and screening, as well as effect size estimation. Additionally, while we also acknowledge the value of open data, there are instances where sharing data may not always be feasible or may not necessarily enable meta-analysts to retrieve the information needed for research synthesis. Therefore, unlike Chow et al. (2023), we place special emphasis on reporting readily available relevant statistics to streamline the meta-analyst's workflow and enhance clarity for all report users.

Through the remainder of this document, we outline the stages associated with conducting a meta-analysis, focusing particularly on those stages directly influenced by the quality of reporting in primary studies. At each stage, we highlight the essential components that need to be incorporated into primary studies to enable future meta-analyses. Furthermore, we discuss the significant role that open science practices play in incorporating a specific study into a research synthesis. Ultimately, we present the Study Eligibility for Meta-Analysis Inclusion (SEMI) checklist, offering concise and clear reporting guidelines for applied researchers to enhance the potential inclusion of their studies in a meta-analysis.

## Searching and screening the literature

In general, a meta-analysis commences with a systematic literature search. Researchers select a set of keywords to search electronic databases for relevant studies. The omission of a crucial keyword may result in overlooking valuable studies in the meta-analysis (Alexander, 2020). The selection of these keywords thus holds substantial significance as it directly influences the number of studies retrieved and may induce bias in the meta-analytic dataset.

In an ideal scenario, meta-analysts would conduct an extensive search for the keywords throughout the full text of research papers. However, if the scope of the meta-analytic investigation or the keyword list is broad, an overwhelming quantity of potentially relevant studies may surface, and many of them may prove irrelevant. To streamline the search process, a commonly employed strategy involves restricting the keyword search to the study title and abstract, assuming that authors normally indicate the most pertinent information within these sections. In this regard, our first recommendation is that authors always clearly mention the most relevant variables under investigation and study characteristics in the study title and abstract so that their study can be easily located during the search phase (aligning with APA reporting standards, see Appelbaum et al., 2018).

Another approach to identifying pertinent studies involves a backward search, wherein references cited within studies are examined. Primary studies, which effectively provide a general overview of the most important literature on the topic and extend beyond the studies published or indexed in databases, serve as a valuable source of studies for meta-analysis. They contribute to the discovery of additional relevant studies that may not have been initially located through conventional database searches, preventing potential oversights in the search process.

Once the researcher has compiled a list of all potential studies, a subsequent step involves the initial screening phase. Based on predetermined inclusion criteria, the researcher (or a group of researchers) assesses the relevance of studies based on their titles and abstracts, excluding those that do not meet the criteria. To expedite the screening process, the title should be as informative as possible and an abstract should capture essential details about a study, offering an accurate record of its conduct and results within the space constraints of a journal (Appelbaum et al., 2018; Polanin et al., 2019). In cases where the title and abstract do not conclusively establish a study's relevance, a meta-analyst is compelled to delve into the full text. Thus, a clear presentation of research objectives or questions and research outcomes within the study is crucial for a swift determination of its relevance.

## Coding the literature

Upon selecting the studies for inclusion in the research synthesis, the next step entails extracting the pertinent qualitative and quantitative information from each study. This information serves three primary purposes: (1) qualitatively summarizing the characteristics of the included studies, (2) quantitatively calculating the desired effect sizes, and (3) conducting moderator analyses, wherein study characteristics (referred to as moderator variables) are employed in a meta-regression model to examine their relationship with the observed effect sizes.

One common challenge in this phase is the incomplete reporting of study characteristics and/or insufficient data within studies to compute the effect size (Lee & Beretvas, 2022; Pigott, 2019; Tipton et al., 2019), which can lead to study exclusion from the meta-analysis (or from moderator analyses) and consequently impact statistical power. Hence, we urge researchers to follow the next guidelines and report study characteristics and outcomes in sufficient detail so that these aspects can be easily coded and used in future research synthesis.

To identify specific study characteristics relevant to future research synthesis, particularly for moderator analyses, the PICO framework (McGowan et al., 2016) can be employed. In this framework, P refers to participant characteristics (e.g., number, age, gender, or socioeconomic status), I refers to intervention or exposure details (e.g., experimental condition, modality, duration, or medication type), C refers to comparator characteristics (e.g., control condition such as a traditional treatment or waitlist), and O refers to outcome characteristics (e.g., a comprehensive description of dependent variables). Alternative frameworks, such as SPICE (setting, perspective, intervention, comparison, evaluation; Booth, 2006) and SPIDER (sample, phenomenon of interest, design, evaluation, research type; Cooke et al., 2012), can also be applied across various study designs.

Within all these frameworks, it is recommended to provide a comprehensive and accurate description of the sample, particularly highlighting characteristics that may impact the results. These include details such as the number of participants identifying as men and women, mean age (including standard deviation or range), region of origin, or socioeconomic status. For research synthesis purposes, authors are urged to present this information on the final analyzed sample, specifically after dropout removal, which may vary across analyses within the same study. Additionally, citing other studies utilizing the same sample or subset thereof is vital to preventing overrepresentation and ensure the unique contributions of samples in meta-analyses. Furthermore, avoiding duplication of samples in meta-analyses is essential for maintaining statistical independence among studies, which is crucial for accurate meta-analytic estimates.

These frameworks also emphasize the necessity of appropriately describing independent and dependent variable(s). For independent variables, such as interventions or experimental conditions (as seen in the PICO or SPICE framework), primary studies should include crucial information such as the modality and intensity of the intervention/experimental condition, its duration (number of sessions and session duration), and details on any administered drugs and their quantities. In correlational studies, where the independent variable is observed rather than experimentally manipulated, it is imperative to furnish information on how the independent variable is operationalized, measured (including reliability measures calculated on the observed data), and implemented. The same level of detail is essential for the dependent variables. Including these specifics not only increases the likelihood that a study can be included in a meta-analysis but also enables the assessment of its risk of bias.

Methodological characteristics are also crucial for meta-analysts to evaluate the methodological quality of primary studies (Pigott & Polalin, 2020). These include the specific research design (e.g., experimental, quasi-experimental, cross-sectional, or longitudinal), procedural details (e.g., where, how, and when data are collected, and the randomization of participants across groups), and specifics of the data-analytic methods (e.g., significance level, statistical tests, and whether the test is two-sided or one-sided).

In terms of reporting the methodological aspects of a study, applied researchers can utilize relevant risk-of-bias assessment tools for comprehensive reporting (refer to https://www.latitudes-network.org/ for an overview of pertinent risk-of-bias assessment tools, Whiting et al., 2023). For example, the widely used Risk of Bias Tool 2 (RoB2; Sterne et al., 2019) for assessing randomized controlled trials includes items such as evaluating the randomization process and assessing bias due to deviations from intended interventions. Authors of primary studies should accurately describe participant assignment and provide specific details on blinding and potential deviations from intended therapy. Systematically reviewing various items from diverse risk-of-bias assessment tools, available for different research designs, significantly assists applied researchers in providing necessary information for others to assess the quality of their studies.

As previously mentioned, proper reporting of numerical results is essential for calculating commonly used effect sizes. The following section provides a brief overview of the statistical outcomes required for effect size calculations.

## Calculating and combining study outcomes

The next step in a meta-analysis involves calculating an index that summarizes the strength of the effect of interest targeted for meta-analysis. Commonly known as an effect size measure, it is defined as “*a quantitative reflection of the magnitude of some phenomenon that is used for the purpose of addressing a question of interest*” (Kelley & Preacher, 2012, pp. 140). However, we do not recommend simply reporting effect sizes that address the research questions of the primary study. This is because the meta-analyst may be interested in an effect size associated with a different set of variables within that study. For example, consider a study with the aim of investigating the effectiveness of an intervention on two dependent variables: well-being and anxiety symptoms. The authors may report two Cohen’s *d* values summarizing the intervention's effectiveness, successfully addressing the intended effect size in that study. However, the meta-analyst might be interested in the correlation between well-being and anxiety symptoms. Since this correlation might not be the primary focus for the primary authors, it might be overlooked in their reporting, consequently leading to the exclusion of the study from meta-analysis. This exclusion can be avoided if authors are contacted to share the correlation value or if they make the dataset publicly available on an online repository, enabling meta-analysts to calculate any desired effect size related to the studied variables. Another reason why merely reporting effect sizes may not be sufficient for a study to qualify for inclusion in a meta-analysis is that for certain types of effect sizes, different formulas exist (e.g., Cohen’s *d* in correlated samples, more information is given in subsequent sections) that might represent different, incomparable parameters (Lakens, 2013). If the authors do not explicitly specify the formula they employed, the meta-analyst in question will be unable to determine whether the reported effect size is appropriate for the research synthesis.

As a result, primary studies should not only report the primary effect size relevant to their specific research question but also provide the necessary numerical information to facilitate its calculation, including its precision (i.e., sampling variance). Since primary researchers might not know which numerical information future meta-analysts will need for their studies, a significant section of this manuscript outlines guidelines regarding the specific quantitative data that should be reported. This aims to enable future meta-analysts to calculate their desired effect size, thereby facilitating the inclusion of the primary study in research synthesis.

The following sections are organized as follows: Firstly, we discuss the role of open science in research synthesis and associated barriers. Next, we attempt to unpack the information that primary investigators should provide in their papers (either in the main text or in supplementary material) to increase the likelihood that their study will be eligible for research synthesis. Although information on the calculation, reporting, and interpretation of effect sizes can be found elsewhere (e.g., Borenstein, et al., 2021; Cooper et al., 2019; Cumming, 2012; Durlak, 2009; Grissom & Kim, 2005 Lakens, 2013; Olejnik & Algina, 2000; Pek & Flora, 2018; Schmidt & Hunter, 2014; Trusty et al., 2004), in Table 1 we provide a summary of the formulas for calculating popular effect sizes to support the information stated below.

**Table 1:** Formulas for the calculation of commonly used effect sizes and corresponding sampling variance

Effect size measure	Study design	Statistics needed	Effect size formula	Sampling variance
Standardized mean difference	Independent groups design	${\overline{X} }_{1}$, ${\overline{X} }_{2},$ ${S}_{1}^{2}, {S}_{2}^{2},$ ${n}_{1}, {n}_{2}$	${g}_{ig}=\frac{{\overline{X} }_{1}-{\overline{X} }_{2}}{{S}_{pooled}} \bullet \left[1-\frac{3}{4\bullet \left({n}_{1}+{n}_{2}\right)-9}\right]$ where ${S}_{pooled}=\sqrt{\frac{\left({n}_{1}-1\right){S}_{1}^{2}+ ({n}_{2}-1){S}_{2}^{2}}{{n}_{1}+{n}_{2}-2}}$	${S}_{{g}_{ig}}^{2}=\left[\frac{{n}_{1}+{n}_{2}}{{n}_{1}\bullet {n}_{2}}+\frac{{g}^{2}}{2({n}_{1}+{n}_{2})}\right]\bullet {\left[1-\frac{3}{4\bullet \left({n}_{1}+{n}_{2}\right)-9}\right]}^{2}$
	Matched group design (1)	${\overline{X} }_{pre}, {\overline{X} }_{post}, {S}_{difference}$, ${r}_{pre-post}$	${g}_{r{m}_{(1)}}=\frac{{\overline{X} }_{post}-{\overline{X} }_{pre}}{{S}_{within}} \bullet \left[1-\frac{3}{4\bullet \left(n-1\right)-1}\right]$ where ${S}_{within}=\frac{{S}_{difference}}{\sqrt{2 (1-r)}}$	${S}_{{g}_{r{m}_{(1)}}}^{2}=\left(\frac{1}{n}+\frac{{{g}^{2}}_{r{m}_{\left(1\right)}}}{2n}\right)\left(2 \left(1-r\right)\right)\bullet {\left[1-\frac{3}{4\bullet \left(n-1\right)-1}\right]}^{2}$
	Matched group design (2)	${\overline{X} }_{pre}, {\overline{X} }_{post}, {S}_{pre}$,${r}_{pre-post}$	${g}_{r{m}_{(2)}}=\frac{{\overline{X} }_{post}-{\overline{X} }_{pre}}{{S}_{pre}} \bullet \left[1-\frac{3}{4\bullet \left(n-1\right)-1}\right]$	${S}_{{g}_{{rm}_{(2)}}}^{2}=\left[\frac{2(1-r)}{n}\right]\left(\frac{n-1}{n-3}\right)\left[1+ \frac{n{g}_{{rm}_{(2)}}^{2}}{2(1-r)}\right]{\left[1-\frac{3}{4\bullet \left(n-1\right)-1}\right]}^{2}-{g}_{{rm}_{(2)}}^{2}$
	Independent groups with pre- and post-test measures (1)	${\overline{X} }_{1, pre}$, ${\overline{X} }_{2, pre}, {\overline{X} }_{1, post}$, ${\overline{X} }_{2, post}, {S}_{1, difference,}$, ${S}_{2, difference,}$,${r}_{1, pre-post}$*, * ${r}_{2, pre-post}$	${g}_{{igpp}_{(1)}}={g}_{{rm}_{(1)}}^{E}-{g}_{{rm}_{(1)}}^{C}$	${S}_{{g}_{{igpp}_{(1)}}}^{2}={S}_{{g}_{{rm}_{(1)}}^{E}}^{2}+{S}_{{g}_{{rm}_{(1)}}^{C}}^{2}$
Standardized mean difference (cont.)	Independent groups with pre- and post-test measures (2)	${\overline{X} }_{1, pre}$, ${\overline{X} }_{2, pre},$ ${\overline{X} }_{1, post}$, ${\overline{X} }_{2, post}, {S}_{1, pre,}$, ${S}_{2,pre,}$, ${r}_{1, pre-post}$*,* ${r}_{2, pre-post}$	${g}_{{igpp}_{(2)}}={g}_{{rm}_{(2)}}^{E}-{g}_{{rm}_{(2)}}^{C}$	${S}_{{g}_{{igpp}_{(2)}}}^{2}={S}_{{g}_{{rm}_{(2)}}^{E}}^{2}+{S}_{{g}_{{rm}_{(2)}}^{C}}^{2}$
Risk ratio	Prospective/Longitudinal studies	${f}_{1}, {f}_{0},$ ${n}_{1}, {n}_{0}$ (see 2×2 table below)	$RR=\frac{{p}_{1}}{{p}_{0}}$ where ${p}_{1}=\frac{{f}_{1}}{{n}_{1}}$and${p}_{0}=\frac{{f}_{0}}{{n}_{0}}$	${{S}^{2}}_{Ln(RP)}=\frac{1-{p}_{1}}{{p}_{1}\bullet {n}_{1}}+\frac{1-{p}_{0}}{{p}_{0}\bullet {n}_{0}}$
Odds ratio	Cross-sectional	${f}_{1}, {f}_{0},{f}_{2}, {f}_{3}$ (see 2×2 table below)	$OR=\frac{{f}_{1}\bullet {f}_{2}}{{f}_{0}\bullet {f}_{3}}$	${{S}^{2}}_{{\text{log}}\left(OR\right)}=\frac{1}{{f}_{0}}+\frac{1}{{f}_{1}}+\frac{1}{{f}_{2}}+\frac{1}{{f}_{3}}$
Psychometric effect sizes	McDonald’s ω	${\lambda }_{i}$,${\varepsilon }_{i}$	$\omega =\frac{{\left(\sum_{i=1}^{I}{\lambda }_{i}\right)}^{2}}{{\left(\sum_{i=1}^{I}{\lambda }_{i}\right)}^{2}+\sum_{i=1}^{I}{\varepsilon }_{i}}$	No consensus (use e.g., bootstrap)
	Validity correction for unreliability	${r}_{xy}$,${r}_{x{x}{\prime}}$,${r}_{y{y}{\prime}}$	${\rho }_{xy}=\frac{{r}_{xy}}{\sqrt{{r}_{x{x}{\prime}}\cdot {r}_{y{y}{\prime}}}}$	No consensus (use e.g., bootstrap)
	Validity correction for range restriction	$r$,${r}^{u}$	${\rho }^{u}=\frac{r}{a}$ where $a=\frac{r}{{r}^{u}}$	${S}_{{\rho }^{u}}^{2}=\frac{{S}_{r}^{2}}{{a}^{2}}$

${\overline{X} }_{1}$= mean of the first group;${\overline{X} }_{2}$= mean of the second group;${S}_{1}^{2}$= variance of the first group;${S}_{2}^{2}$= variance of the second group;

${n}_{1}$= sample size of the first group;${n}_{2}$= sample size of the second group;${\overline{X} }_{pre}$= mean of the pre-test;${\overline{X} }_{post}$= mean of the post-test;${S}_{difference}$= standard deviation of the difference scores (i.e., difference between pre-test and post-test scores);${S}_{pre}$= standard deviation of the pre-test scores;${r}_{pre-post}$= correlation between pre-test and post-test scores;${\overline{X} }_{1, pre}$= mean score of first group at pre-test;${\overline{X} }_{2, pre}$= mean score of second group at pre-test;${\overline{X} }_{1, post}$= mean score of first group at post-test;${\overline{X} }_{2, post}$= mean score of second group at post-test;${S}_{1, difference}$= standard deviation of the difference scores in the first group;${S}_{2, difference}$= standard deviation of the difference scores in the second group;${r}_{1, pre-post}$= correlation between pre-test and post-test scores in the first group;${r}_{2, pre-post}$= correlation between pre-test and post-test scores in the second group;${\lambda }_{i}$= standardized factor loading for item *i* in 1 to I;${\varepsilon }_{i}$= error variance for item *i* in 1 to *I*;${r}_{xy}$*=* correlation between scale scores and criterion;${r}_{x{x}{\prime}}$= reliability of scale scores;${r}_{y{y}{\prime}}$= reliability of the criterion;$r$: observed validity coefficient;${r}^{u}$= unrestricted validity coefficient;${S}_{r}^{2}$= variance of the observed validity coefficient

### Open science

While comprehensive reporting is crucial for study eligibility in research synthesis as outlined in the following sections, the significance of this reporting may diminish if raw datasets are consistently accessible. If raw datasets are publicly available, meta-analysts could calculate any effect size of interest, whether the one reported in the study or any other beyond the primary study goal. Additionally, with raw data available in all studies, individual participant data meta-analyses (Riley et al., 2010) could be systematically performed. Hence, giving access to the datasets would undoubtedly assist meta-analysts in retrieving important data to conduct a research synthesis, namely the effect sizes and relevant information for the moderator analyses.

Despite the increasing number of journals and granting agencies mandating the sharing of collected data, the actual practice of data sharing remains relatively infrequent. Obstacles to data sharing extend beyond technical challenges. Issues such as the absence of recognition incentives for sharing research data, the absence of standardized formats for data and metadata (that offer the details necessary for other researchers to comprehend the data), privacy concerns, fear of misuse, and limited time and resources all pose potential hindrances to effective data sharing (Krumholz, 2012).

Even in cases of successful data sharing, it does not necessarily contribute to resolving reporting issues for meta-analysis. First, providing the dataset and the analytics code to reproduce the main results does not always ensure reproducibility (Hardwicke et al., 2018; Hardwicke et al., 2021; Obels et al., 2020). This is because authors may make errors in the dataset and/or code, or they may not provide the complete code necessary to reproduce all analyses. Additionally, authors may overlook the inclusion of metadata, hindering the comprehension of variables within the dataset. On top of this, the inadequate reporting of crucial study details, such as the research procedure, sample characteristics, instrument details, and research design, remains unresolved even with the availability of a publicly accessible dataset. In essence, having access to a dataset does not guarantee that meta-analysts will acquire comprehensive information from the study necessary for inclusion in meta-analysis or meta-regression analyses, especially details suitable for moderator analyses. Hence, our recommendation is not only to provide access to the dataset and code used but also to adhere to the guidelines outlined in this manuscript.

When providing public access to the dataset and analytics code, it is crucial to consider specific key factors for ensuring the success of the process (see also Obels et al., 2020; Wilkinson et al., 2016). First, ensure the public accessibility and proper functionality of the website link hosting the documents. Second, provide a comprehensive codebook that clearly explains the coding for each variable. Third, include explanatory comments in the analytical code to guide fellow researchers through its execution. Finally, to overcome interoperability challenges and to ensure compatibility across different statistical software packages and versions, store data in universally readable formats such as .ASCII, .CSV, and .TXT. For comprehensive guidance on the process of data sharing, please refer to the step-by-step guide provided by Logan et al. (2021). This resource offers detailed insights and instructions to help one effectively navigate the various stages of sharing data.

### Univariate statistics of the whole sample

Descriptive summary statistics (e.g., sample sizes, means, standard deviations, frequencies, and proportions) are crucial for accurately describing the variables under study and for calculating the most relevant effect sizes, including standardized mean differences, risk ratios, and odds ratios (see Table 1). It is important to highlight the necessity of providing this information for the final sample of participants after excluding dropouts. In longitudinal studies, providing descriptive statistics for each time point is particularly vital, especially in instances where participants were absent, or data were missing.

When studying qualitative categorical variables, such as dichotomous, nominal, or ordinal variables (e.g., socioeconomic status, type of stimuli, or type of task), frequencies and proportions should be reported for each category of the qualitative variable, regardless of whether it is an independent or dependent variable. For instance, in studies on inattentional blindness—where individuals may fail to notice unexpected stimuli in their visual field due to focused attention on a different task or stimulus—the typical dependent variable is whether individuals notice an object unexpectedly introduced by the researcher in the task (e.g., Wiemer, et al., 2013), and authors should report the number and proportion of individuals who noticed the unexpected objects and those who did not.

Moving on to quantitative variables (e.g., age, income, or test scores), the descriptive statistics to be reported are means and standard deviations.^1^ For instance, Harris (2004) examined the relationship between intelligence, achievement, openness to experience, and creativity. All these variables were quantitatively measured, and their means and standard deviations are appropriately presented in a table. Harris (2004) did not specify whether there was missing data, leading to the assumption that all variables are based on the complete sample. Ideally, it should be explicitly mentioned that no data were missing, or the sample size for each variable could have been indicated. Another instance is the study conducted by Goecke et al. (2020), where they investigated conflicting assertions regarding the overclaiming phenomenon (i.e., the inclination of individuals to overrate both their general cognitive abilities and their specific knowledge). The researchers measured various quantitative variables, including overclaiming, self-reported knowledge, and crystallized intelligence, and detailed their means, standard deviations, and corresponding sample sizes in a table. Notably, they provided precise information about the sample for each variable, with slight variations in sample sizes due to missing data. This meticulous reporting enables a future meta-analyst to discern the exact sample for each of these measures.

### Descriptive statistics for the relationship between variables

When examining the relationship between variables, it is important to report the descriptive information associated with this relationship because this is the information commonly used by meta-analysts to calculate effect sizes. In the following subsections, we disaggregate this information by the types of variables involved in the relationship.

#### Relationship between categorical variables

The numerical information required for studying the relationship between categorical variables depends on the type of categorical variables under investigation. When studying the relationship between two dichotomous or nominal variables, it is imperative to present a cross-tabulation with disaggregated frequencies. Such cross-tabulation provides the necessary information to calculate effect sizes, such as odds ratios and risk ratios, which are commonly used in meta-analyses of categorical data. For instance, consider a study investigating the association between smoking status (smoker vs. non-smoker) and the presence of lung cancer (yes vs. no). A cross-tabulation of these variables would display the frequencies of individuals falling into each combination of categories, for instance, the number of smokers diagnosed with lung cancer, non-smokers diagnosed with lung cancer, smokers not diagnosed with lung cancer, and non-smokers not diagnosed with lung cancer (see, for example, Morabia & Wynder, 1991). This detailed breakdown is essential for meta-analysts aiming to synthesize the association between these two variables across studies.

When studying the relationship between a dichotomous or a nominal variable and an ordinal variable or between two ordinal variables, it is crucial for researchers to provide access to the dataset containing raw data. In other words, if researchers utilize ordinal variables and aim for their study to be eligible for future meta-analyses, adherence to open science practices is imperative. This is because most effect sizes applicable to ordinal variables cannot be computed solely from descriptive summary statistics. For instance, to assess the magnitude of the difference between two groups in an ordinal variable, one might calculate the delta Cliff (Cliff, 1993), but raw data are indispensable (see Macbeth et al., 2011). Similarly, the correlation between two ordinal variables can be determined using Spearman or Kendall’s tau-square correlation (Kendall, 1938), but once again, raw data are necessary for computation, as it involves examining concordant and discordant pairs of observations. Consequently, meta-analysts interested in effect sizes related to ordinal variables can include a particular study in their research synthesis only if the exact effect size of interest is reported or if authors have made their datasets publicly available.

#### Relationship between categorical and quantitative variables

When investigating the relationship between a categorical variable and a quantitative variable, means and standard deviations of the quantitative variable should be reported for each category of the categorical variable. Harris' (2004) study provides an example of how descriptive statistics for quantitative dependent variables are reported by pertinent groups. In this investigation, gender differences were examined, and a breakdown of means and standard deviations segregated by gender is provided in a table. This detailed presentation of descriptive statistics for relevant subgroups, such as based on gender, aids future meta-analysts in computing standardized mean differences between genders across all measured variables. Especially in studies where the primary analysis involves an analysis of variance (ANOVA), it is crucial to report means, standard deviations, and sample sizes for each combination of categories of the qualitative variables used as the independent variable in the analyses. For instance, consider a two-factor ANOVA with independent variables such as socioeconomic status (low, medium, and high) and educational level (primary, high school, and university). In this case, means, standard deviations, and sample sizes should be reported for each of the 3 × 3 = 9 subgroups resulting from the combination of categories. This detailed reporting is essential as it enables meta-analysts to calculate standardized mean differences for any of the resulting subgroups. It is important to note that this descriptive information should be reported regardless of the primary researcher's specific focus, which typically revolves around the interaction between the independent variables, and it does not necessarily have to be included in the main text; it can be relocated to the supplementary materials.

#### Relationship between quantitative variables

Pearson correlation coefficients summarize the (linear) relationship between two quantitative variables. These coefficients are incredibly useful in meta-analysis for several reasons. Firstly, correlation coefficients serve as effect sizes that can be readily integrated into meta-analytic datasets. Second, many partial effect sizes can be calculated from correlation coefficients, such as partial- and semi-partial correlations and standardized regression coefficients (Aloe & Becker, 2009, 2012; Becker, 1992; Fernández-Castilla et al., 2019). However, if researchers only report the results of multiple regression models (i.e., unstandardized or standardized regression coefficients), correlation coefficients cannot be back-calculated,^2^ and this is a reason why many primary studies are often discarded for meta-analysis. Although a procedure to convert regression coefficients to correlations has been proposed (Peterson & Brown, 2005), it does not work correctly in many scenarios (Aloe, 2015). Hence, simply reporting correlations among quantitative variables enables the calculation of many effect sizes that might be of interest to meta-analysts.

A third reason why correlation coefficients should always be reported is that, to implement multivariate meta-analytic models, the correlation between the raw scores of the variables of interest is needed. For instance, imagine that a meta-analyst is interested in synthesizing standardized mean differences that reflect the effectiveness of a given psychological intervention in reducing both anxiety and depressive symptoms, and that most studies report these two results. Since there are two correlated dependent variables within studies (anxiety and depression), a multivariate meta-analysis would have to be carried out to synthesize these effect sizes (Becker, 2000; Kalaian & Raudenbush 1996). To conduct this type of analysis, the covariance between the standardized mean differences reported in the same study (presumably one for depression and one for anxiety) needs to be estimated in advance (see Hedges & Olkin, 1985), and to calculate it, information on the correlation between the raw depression and anxiety scores is needed. By reporting the correlation coefficients between all quantitative variables, future meta-analysts will be able to retrieve this information to apply more sophisticated statistical methods, eventually leading to more precise meta-analytic estimates.

A final reason why reporting correlation coefficients is important is that new methods have been developed in the field of meta-analysis, such as meta-analytic structural equation modeling (also known as MASEM, Cheung, 2015; Jak, 2015; or one-stage MASEM [OSMASEM], Jak & Cheung, 2020). This methodology allows one to perform meta-analysis of more complex structural equation models, including mediation models (e.g., Ng et al., 2023), path analyses (Smith et al., 2022), or confirmatory factor analyses (Said-Metwaly et al., 2018). The input required for conducting MASEM is the correlations between the variables of interest organized in a correlation matrix. By reporting all the possible correlations of one’s dataset in a correlation matrix, meta-analysts performing MASEM could easily include all the correlations between their variables of interest.

#### Intraclass correlation coefficient and variance estimates in cluster-randomized studies

In primary research within the realms of psychology and educational sciences, it is commonplace to encounter hierarchical structures wherein observations are nested within higher-level clusters. Examples include students nested within classrooms or observations nested within participants in repeated measures designs. This hierarchical structure necessitates consideration not only during data analysis but also in the calculation of certain effect sizes, such as the standardized mean difference (Hedges, 2007; Snijders, 2005).

Consider, for instance, a scenario where two groups of participants from distinct experimental conditions are compared (level 1), and these participants are further grouped into different centers, forming the cluster at level 2. When calculating the standardized mean difference that compares means across these experimental conditions, it becomes imperative to acknowledge that participants are nested within different clusters (centers in this case). Consequently, participants belonging to the same center are expected to exhibit greater similarity than those from different centers.

There is no singular formula for calculating a standardized mean difference for clustered designs. The mean difference between groups may be standardized by the square root of the pooled within-cluster variance, the between-clusters variance, or the total variance, representing the sum of the two variances. Therefore, to facilitate the calculation of any of these versions of effect sizes, a meta-analyst must possess information on (1) the mean of the two compared groups, (2) the between-clusters variance, and (3) the within-cluster variance. These sources of variability can also be estimated from each other if the intraclass correlation coefficient is available. This coefficient signifies the correlation between observations within the same cluster, and the relevant formulas can be found in Borenstein and Hedges (2019). The intraclass correlation coefficient, coupled with the total sample size and average cluster size, is also essential for calculating the sampling variances of these effect sizes. Consequently, it is of utmost importance to thoroughly report all this information in studies employing such designs.

#### Pearson correlations between repeated measures

In meta-analysis, it is often of interest to include data from matched group experimental designs meant to test the effectiveness of an independent variable (e.g., intervention, program, or experimental condition). Typically, in each study, standardized mean differences for repeated measures (see $${g}_{rm(1)}$$ in Table 1) or standardized mean changes (see $${g}_{igpp(1)}$$ in Table 1) are calculated for posterior synthesis. Importantly, the formulas for these effect sizes incorporate the correlation between pre- and post-measures. Specifically, this correlation is essential for determining the standard deviation of the difference ($${S}_{within})$$, which serves as the denominator in the formula for computing the standardized mean difference for repeated-measures designs ($${g}_{rm(1)}$$ in Table 1). Furthermore, this correlation between pre- and post-test scores is necessary for calculating the sampling variance of this effect size (see Morris and DeShon, 2002). Similarly, to calculate a standardized mean change (i.e., the standardized difference in the extent of change within one group relative to the change observed in another group, see $${g}_{igpp(1)}$$ in Table 1), the correlation between pre- and post-measures within each involved group is also required.

Since authors seldom report this correlation, formulas have been proposed to circumvent its inclusion in the calculation of these effect sizes (see, for instance, Becker, 1988; see formula for ${g}_{rm(2)}$ and ${g}_{igpp(2)}$ in Table 1). However, this pre/post score correlation is still essential for computing the sampling variance of these effect sizes (see ${S}_{{g}_{rm(2)}}^{2}$ and ${S}_{{g}_{igpp(2)}}^{2}$ in Table 1). Therefore, when utilizing standardized mean differences or standardized mean changes in meta-analysis, the correlation between pre- and post-measures often needs to be estimated or imputed. Hence, we strongly encourage primary researchers to incorporate this correlation in their reports, along with any other pertinent descriptive information.

#### Reliability of the measurements

Reliability is commonly defined as the proportion of true score variance to total score variance (Novick, 1966). Reliability coefficients provide information on the precision of scores from psychological assessments. In psychological science, measurements frequently contain non-negligible degrees of error. For instance, self-reported outcomes may include nuisance related to the distortions in individuals’ self-perception or understanding of the response scale. These measurement errors are generally assumed to be random variations that cause scores to deviate from their true values.

Although often disregarded, the results of a primary study containing psychological assessments are largely influenced by measurement reliability. As an example, in the relationship between general intelligence and job performance, if both measures are precise (e.g., obtained using long questionnaires), the estimated regression/correlation coefficients are likely to approximate the true relationship between these constructs. On the other hand, if measurement reliability is low (e.g., using fewer or more imprecise questions), the coefficients between variables may be largely underestimated. To illustrate, a correlation of 0.51 between intelligence and job performance (e.g., Schmidt & Hunter, 2004) could be substantially reduced, to approximately 0.36, if both measurements have reliability coefficients of around 0.70.

Meta-analytic studies are often aimed at summarizing generalized coefficients for the relationships between constructs beyond one specific sample. Correcting these underestimated regression/correlation coefficients relies on the reliability indices reported in primary studies. Authors are encouraged to report reliability coefficients (e.g., Cronbach's ɑ or McDonald's ⍵) of their measurements. Finally, it is important to note that the reliability reported in an instrument’s manual or in the original validation studies may not precisely match reliability in empirical studies. Due to range restrictions of the scores and additional noise due to various random factors, the reliability in an empirical study can differ from the one in the original validation study. Hence, authors of primary studies are encouraged to report the reliability of measurements obtained in their datasets. This not only enhances their reporting but also makes their studies eligible for future reliability generalization meta-analysis.

### Negative results

Researchers may conduct a study and find an effect that is either statistically nonsignificant or contradicts a hypothesis, referred to as a negative finding. Negative findings face a greater publication challenge than their positive counterparts (Fanelli, 2010; Franco et al., 2014). Researchers may fuel publication bias by selectively reporting positive findings or refraining from submitting studies with negative findings. This behavior is often driven by the anticipation of low acceptance rates, or the fear of professional consequences associated with publishing findings that challenge well-confirmed hypotheses or theories (Shields, 2000; Therrien & Cook, 2018). Journal editors and reviewers may also contribute to publication bias by rejecting submissions with null findings.

Publication bias has been observed in various fields, including medicine, social sciences, and psychology, indicating a widespread phenomenon (Therrien & Cook, 2018). Publication bias may inflate the estimates of relationships between variables and treatment effects in meta-analyses. The inclusion of even a few unpublished findings could substantially influence conclusions drawn from the literature (Howard et al., 2009; Polanin et al., 2016). Publication bias distorts scientific literature, leading to the formulation of hypotheses or taking decisions in practice based on inaccurate information, wasting research opportunities and funding and violating an implicit contract with funders (Shields, 2000). Moreover, when negative findings go unpublished, researchers may expend resources conducting studies that have already proven unsuccessful (Fanelli, 2012). The potential bias in the literature, however, is not the only problem with not reporting findings. We also have an ethical responsibility to our study participants who invest their valuable time and resources, trusting that their contributions benefit others. Failure to publish study findings violates this trust and may be deemed scientific misconduct (Chalmers & Moher, 1993; Shields, 2000). Additionally, we owe transparency to donors and taxpayers who support our research.

To encourage the publication of negative findings, it is crucial to recognize the value of negative results on par with positive ones. Acknowledging that understanding the absence of an effect holds equal importance to identifying its presence is essential (Fox & Kaufman, 2018). Instead of planning studies solely to determine "what works," a shift to planning studies to understand "how to make things work better" allows for useful insights from positive or negative findings (Jacob et al., 2019). By shifting our perspective and acknowledging the importance of negative findings, we contribute to a more balanced and comprehensive scientific literature, fostering a culture that appreciates the diverse outcomes of rigorous research efforts.

Recognizing their significance, initiatives have been undertaken to improve the visibility of negative findings in scientific literature through diverse approaches. For instance, certain journals have been initiated exclusively dedicated to publishing negative findings, such as the *Journal of Negative Results*, *Journal of Negative Results in BioMedicine*, *Journal of Pharmaceutical Negative Results*, *Nature's* Negative Results section, and *Positively Negative (PLOS One)*. In addition, mainstream journals have allocated special issues specifically for null findings (see, for instance, Landis et al., 2014; Therrien & Cook, 2018). However, this approach may inadvertently introduce bias favoring negative outcomes (Mlinarić et al., 2017). Publishing criteria should thus prioritize study quality and statistical power, irrespective of the direction and significance of the results.

Journal editors and reviewers can also play a pivotal role in shaping positive attitudes and behaviors regarding negative findings. For instance, editors can explicitly express in the author guidelines the openness to publish well-designed studies with null findings (Hubbard & Armstrong, 1992). Editors can also promote or mandate registered reports, where study plans are submitted for pre-publication review based on research design. If accepted, the study is published regardless of the reported findings, minimizing the likelihood of result-driven deviations or studies being overlooked in file drawers (Cook & Therrien, 2017). Moreover, during the revision process, editors and reviewers commonly ask for the removal of information that is deemed nonessential, frequently tied to negative findings. While brevity is important, we should not sacrifice information. Unless entirely unrelated to the primary research question, it is advisable to report findings regardless of their direction, even if placed in supplementary material—thus, providing more information is generally preferable (Landis et al., 2014). Such practices could address publication bias by directly publishing more studies with negative findings and indirectly affirming their value and publishability, encouraging researchers to submit rather than keep them in a file drawer (Cook & Therrien, 2017).

## Study Eligibility for Meta-Analysis Inclusion (SEMI) checklist

Many reporting guidelines have been provided for studies of different fields: the STROMS checklist for research on human microbiome (Mirzayi, et al., 2021), the AGREE Reporting Checklist for clinical research (Brouwers et al., 2016), and the CROSS checklist for survey studies (Sharma et al., 2021). In this direction, some interesting initiatives have emerged, such as the EQUATOR Network (Altman et al. 2008), which brings together different resources and checklists that aim to improve the accuracy of the reporting and the quality of the research. There are also well-known reporting guidelines developed to properly report information in meta-analyses and systematic reviews (the PRISMA statement, Page et al., 2021; the REGEMA checklist, Sánchez-Meca et al., 2021 in reliability generalization meta‐analyses). However, there is currently a lack of reporting guidelines specifically aimed at enhancing the odds of a study being retrieved and being eligible for a meta-analysis, and that is the gap aimed to be filled with this manuscript.

In this section, we provide the SEMI checklist to supplement extant reporting guidelines in the hope of improving the completeness of information in primary empirical reports and thus optimizing for inclusion in future meta-analyses. The SEMI checklist may be used in conjunction with other checklists assessing basic reporting prerequisites (e.g., PRISMA, STROBE, and CONSORT), aiming to maximize the quality of reporting practices and facilitate accumulated meta-analytic knowledge.

The development of the checklist items was informed by existing reporting guidelines, our own experience in meta-analysis research, and consultation with expert researchers in the field. It was also guided by the PICO model, which is frequently used for planning literature search and study selection in research synthesis (McGowan et al., 2016).

Initially, The SEMI checklist involved 30 items, followed by a “yes/no/not applicable” judgment, covering five key parts of a paper: title and abstract, background, methods, results, and open science. We incorporated items related to study title and abstract to encourage researchers to consider reporting information that support meta-analysts to retrieve the study in database searching and to conduct title/abstract screening. We also incorporated items related to a study background to facilitate locating relevant studies via backward reference searching. In the Methods section, we present elements pertaining to the accurate reporting of study characteristics, crucial for the subsequent execution of moderator analyses in meta-analysis. Next, in the Results section, our focus is on elements related to the proper reporting of numerical information, essential for calculating effect sizes. We also incorporated items to prompt researchers to report results in a sequence that mirrors the description of analyses outlined in the Methods section and to ensure coherence between the textual results with those displayed in the tables and figures. This can help mitigate ambiguity and potential misinterpretation, offering meta-analysts a clear roadmap to navigate the study's design, methods, and results without unnecessary confusion. Finally, we also include some items related to open science practices. This initial version of the checklist underwent review by four external methodological experts in the field of meta-analysis, some with more than two decades of experience in meta-analysis, who provided valuable feedback to refine the tool. Incorporating expert opinions, we revised existing items and introduced new ones, resulting in a final set of 28 items.

We make the SEMI available in Table 2 for the research community and will register it on the EQUATOR website to enhance dissemination. We recommend journals and publishers endorse the use of the SEMI by referring to it in their instructions to authors and consider utilizing it in their review process.

**Table 2:** Study eligibility for meta-analysis inclusion checklist

Item	Y	N	NA
Title and abstract			
1. The key concepts, constructs, and variables under investigation are clearly mentioned in the title and/or abstract.			
2. The abstract gives relevant details about the study objectives, methods, and results.			
Background			
3. Relevant literature (including reviews and meta-analyses) is summarized and clearly cited.			
Methods			
4. The sample size, including that of the entire sample and each subsample, is reported. The number of missing values is given for each variable, and the sample size used for each analysis is reported. In the case of longitudinal studies, the sample size at each time point is reported.			
5. Statistics describing participant characteristics (e.g., proportion identifying as men, mean age, proportion of sample by race/ethnicity), study context and procedures that may (substantially) influence the studied effects are reported.			
6. Other publications based on the same data, or a portion thereof, are clearly cited.			
7. There is a description of how each variable is operationally defined and measured.			
8. Details of how the measurement tools are administered and scored are provided, together with a measure of reliability on the current sample.			
9. Details of the type of study design (e.g., correlational, comparative, or experimental) are provided, possibly together with a bibliographic source for further details.			
10. Details of the study procedures are provided, including where, how, and when data are collected.			
11. There is a description of how data categories are defined or how continuous variables are categorized. When reporting data from a subsample, details on subsample descriptions and selection criteria subsample are provided.			
12. Details of the data-analytic methods used are provided (e.g., statistical tests, model fitted, estimation procedure, software, options chosen, significance level, whether the test is two-sided or one-sided, degrees of freedom, how cluster data are handled if needed, and whether missing data imputation methods were used and which ones).			
13. A risk of bias assessment tool is consulted to ensure the inclusion of all methodological details required for evaluating the study's risk of bias.			
Results			
14. For categorical variables, frequencies of all categories are reported for the final sample and relevant subgroups, after removing dropouts. When studying the association between categorical variables, a cross-tabulation is provided with disaggregated frequencies.			
15. For quantitative variables, means and standard deviations are provided for the whole sample and relevant subgroups.			
16. For nested data structures, information on the intraclass correlation coefficient, the between-clusters variance, and the pooled within-cluster variance is reported.			
17. The correlation matrix between all quantitative variables under investigation is reported. When missing data are imputed, correlations based on the original incomplete data are provided.			
18. For longitudinal studies, the timing of measurements and the correlation between subsequent measures is reported, also for any relevant subgroup.			
19. Test statistics and associated *p*-values (and degrees of freedom where relevant) are reported, also for negative findings.			
20. Effect sizes related to the research questions are presented along with (references to) the corresponding formulas used for their calculation.			
21. The results are reported in sufficient detail and clarity, following the description of the analyses in the methods-section (e.g., following the same order).			
22. The results presented in the text align with those depicted in the tables and figures.			
23. Tables and figures are appropriately labelled, understandable and referred to in the text.			
Open science practices			
24. A statement indicating the availability and location of raw study data (and if applied of the protocol or registered report) is provided.			
25. If a protocol or registered report was developed before the investigation, it is clarified how the investigation deviates from the initial planning.			
26. A code book explaining the variables in the dataset is provided.			
27. Relevant codes/syntax that reproduce the analyses are provided.			
28. Additional information or materials that could enhance understanding of methods or results are included in appendices or supplementary materials.			

Y = Yes; N = No; NA = Not applicable

## Discussion

Meta-analysis has emerged as a powerful tool for consolidating scientific knowledge and informing decision-making. However, the accurate execution of various stages of a meta-analysis may be hindered by the inaccurate reporting of information in primary research studies. If studies cannot be found or if effect sizes cannot be computed, they will be excluded from the research synthesis, ultimately impacting the statistical power to detect a significant overall effect or even inducing bias. Likewise, if the characteristics of the studies cannot be effectively encoded, there will be missing information in the moderator analysis, which in turn will affect the analytical power (Pigott, 2019). Although imputation techniques exist to prevent this problem (e.g., Lee & Beretvas, 2022), no technique will yield the same accurate estimates as having all the data available for the analyses.

For this reason, we have introduced the SEMI checklist, which can be utilized to assess the suitability of a study for inclusion in future meta-analyses. To the best of our knowledge, this checklist represents one of the first endeavors to improve the reporting quality of primary studies, with a specific focus on their potential inclusion in a meta-analysis. In a similar vein, Chow et al. (2023) have offered valuable recommendations for reporting specific elements of studies, such as procedures, results, and open access practices. Our checklist broadens the scope of Chow’s checklist to include additional critical elements. This encompasses aspects such as the study's title, abstract, background, sample characteristics, and other results essential for calculating various effect sizes in meta-analysis, thereby ensuring a more comprehensive reporting framework.

Hopefully, the use of the SEMI checklist and the Chow et al. (2023) guidelines can assist authors in describing the conducted research in sufficient detail, assist editors and reviewers in evaluating the comprehensiveness of reports submitted for publication, and ultimately maximize the use of research results in the quantitative synthesis. We believe that adhering to the suggested checklist can substantially enhance the reporting standard of primary studies. This, in turn, will ultimately contribute to conducting more precise and reliable meta-analyses.

## Footnotes

1 Common analyses (e.g., regression analyses) assume that the residual scores on the outcome variable are normally distributed. A violation of this assumption or the existence of outliers may make means and standard deviations less informative. Therefore, information on the distribution of the scores is also required.

2 An exception would be the method presented by Fernández-Castilla et al. (2019), where correlation coefficients can be estimated from standardized regression coefficients under some scenarios.
